# Toxic Markers of Matrine Determined Using ^**1**^H-NMR-Based Metabolomics in Cultured Cells *In Vitro* and Rats *In Vivo*

**DOI:** 10.1155/2015/598412

**Published:** 2015-08-30

**Authors:** Zhonghuang Li, Liang Zheng, Jian Shi, Guiyu Zhang, Linlin Lu, Lijun Zhu, Jiajie Zhang, Zhongqiu Liu

**Affiliations:** ^1^School of Pharmaceutical Sciences, Southern Medical University, Guangzhou, Guangdong 510515, China; ^2^International Institute for Translational Chinese Medicine, Guangzhou University of Chinese Medicine, Guangzhou, Guangdong 510006, China

## Abstract

Matrine is one of the main bioactive alkaloids of *Sophora flavescens* Aiton, which has been widely used to treat various diseases in China. These diseases include viral hepatitis, liver fibrosis, cardiac arrhythmia, skin diseases, and tumors. However, matrine is also the main toxic compound of this herb, and the available biomarkers are not reliable in detecting or quantifying matrine risk. Metabolomics is a powerful tool used to identify early toxicity biomarkers that are specific indicators of damage to biosystems. This study aimed to find the potential biomarkers of the matrine-induced toxic effects in rats and HepG2 cells. The toxicological effects of rats induced by matrine could be derived from the elevated taurine and trimethylamine N-oxide levels and the depletion in hippurate and tricarboxylic acid cycle intermediates, such as 2-oxoglutarate, citrate, and succinate in the urine. Cell metabolomics revealed that the levels of alanine, choline, glutathione, lactate, phosphocholine, and cholesterol showed dose-dependent decreases, whereas the levels of taurine, fatty acid, and unsaturated fatty acid showed dose-dependent increases. Overall, a significant perturbation of metabolites in response to high dose of matrine was observed both *in vivo* and *in vitro*, and the selected metabolites particularly represent an attractive marker for matrine-induced toxicity.

## 1. Introduction

Matrine is one of the main bioactive alkaloids derived from the root of* Sophora flavescens* Aiton, which has been used in traditional Chinese medicine for its antiallergy, anti-inflammatory, and antiviral properties [1–3]. Recent evidence has indicated that matrine has potent antitumor activities, such as inhibiting cancer cell proliferation, restraining angiogenesis, reversing multidrug resistance, and preventing or reducing chemotherapy or radiotherapy toxicity when combined with other chemotherapy drugs [4, 5], and thus is considered as a promising drug for cancer therapy [6]. However, the possible toxicity of matrine remains a concern. Several studies in rodents have suggested that the toxic targets of matrine are the nervous system and liver [7–9]. Clinical cases also reported that oral administration or injection of a high dose of matrine results in liver toxicity, cardiac side effects, and neurological abnormality in patients [10, 11]. However, the effect of matrine on endogenous metabolites of animals and cells has not been reported. The possible toxicity mechanisms and risks have not been fully elucidated. Therefore, systematical evaluation of matrine toxicity is still necessary.

Metabolomic technologies enable monitoring of endogenous small molecule metabolites, revealing the ultimate response of a biologic system to genetic factors and/or environmental changes [12]. Metabolomics is also a powerful tool to investigate toxicity by monitoring the metabolic profile of biofluids, tissues, and cells, which contain a mixture of metabolites. The abnormality variations of biomolecules in biofluids, also known as biomarkers, induced by drugs are extracted to improve the understanding of the disease or toxic mechanisms [13, 14]. Various analytical platforms, such as nuclear magnetic resonance (NMR), gas chromatograph-mass spectrometry (GC/MS), and liquid chromatograph-mass spectrometry (LC/MS), have been developed to qualitatively and quantitatively detect metabolites [15]. NMR spectroscopy is an information-rich analytical technique that provides comprehensive chemical information about the composition of biological samples. NMR-based metabolomic analysis has been successfully applied to identify the novel biomarkers for drug toxicity studies. Various biomarkers based on metabolomics have already been found for prediction of drug-induced harmful toxic effects. For instance, drug-induced renal papillary damage isassociated with altered levels of dimethylamine, trimethylamine N-oxide (TMAO), N,N-dimethylglycine, and succinate in urine [16]. Meanwhile, urinary endogenous metabolites of TMAO, citrate, 3-chlorotyrosine, phenylalanine, glycine, hippurate, and glutarate as well as plasma endogenous metabolites of lactate, glucose, 3-hydroxyisovalerate, isoleucine, acetylglycine, acetone, acetate, glutamine, ethanol, and isobutyrate significantly respond to APAP dosing in humans [17].

In this work, an NMR-based metabolomic approach was applied to carry out preclinical toxicological investigation of matrine using* in vivo* (Sprague-Dawley rats) and* in vitro* (HepG2 cells) experimental models to evaluate its potential toxicity. ^1^H-NMR spectroscopy coupled with multivariate statistical analysis was used to characterize the metabolic profiles of rat urine and HepG2 cells after treatment with matrine. Compared with other conventional methods, this study detailedly detected the differential endogenous metabolites as the available biomarkers to characterize the matrine-induced toxicity in SD rats and HepG2 cells and to provide elementary data for clinical administration and relative future research.

## 2. Materials and Methods

### 2.1. Chemicals and Reagents

Matrine was purchased from Chengdu Mansite Pharmaceutical Co. Ltd. (Chengdu, China). D_2_O, chloroform-D, sodium azide, and 3-(Trimethylsilyl) propionic-2,2,3,3-d_4_ acid sodium salt (TSP) were acquired from Sigma-Aldrich (St. Louis, MO, USA). Pure water was obtained from Millipore Alpha-Q water system (Bedford, MA, USA). Fetal bovine serum and penicillin-streptomycin solution were purchased from Thermo Fisher Scientific (MA, USA). All other reagents used were of analytical grade or better and used as received.

### 2.2. Animals

Twenty male SD rats (5 weeks old, body weight of 150–180 g) were purchased from the Medicine Laboratory Animal Center of Guangdong province. The rats were acclimated for 1 week in an environmentally controlled room at 24 ± 1°C on a 12 h light/dark cycle and provided with food and water ad libitum. The surgical procedures and experimental protocol were approved by the Guidelines for Animal Experimentation of Southern Medical University.

### 2.3. Drug Administration and Sample Collection

The rats were randomly divided into four groups (five rats each group) and were treated with matrine at the dose of 0 (control), 30 (low dose), 60 (middle dose), and 90 mg/kg (high dose) body weight. Matrine was dissolved in ultrapure water with a concentration of 3, 6, and 9 mg/mL. All administration groups received a single dose of matrine via oral gavage, and control group was similarly provided with an equal volume of ultrapure water.

Urine samples were collected into tubes containing 1 mL of 1% sodium azide overnight on days 1 and 3 after dosing. The collected urine was immediately centrifuged at 10000 g for 10 min to obtain a clear suspension and was stored at −70°C until required for NMR experiments.

### 2.4. Cell Culture and Drug Treatments

Cells were cultured in DMEM supplemented with 10% (v/v) fetal bovine serum, 1% sodium pyruvate, 2 mM glutamine, 100 *μ*g/mL streptomycin, and 100 U/mL penicillin at 37°C in a humidified atmosphere of 5% CO_2_. Cells were seeded at a density of 3.0 × 10^5^ cells/75 cm^2^ flask in 12 identical sets (five samples each group) and treated with different concentrations of matrine (0, 0.2, 0.4, and 0.7 mg/mL) for 12, 24, and 48 hours. Then, the cells were harvested through trypsinization with 1 mL of trypsin solution and washed twice with ice-cold PBS (4°C, pH 7.4). All samples were rigorously treated with the same procedure to minimize experimental variability. The cell samples collected were immediately snapped frozen in liquid nitrogen and stored at −70°C until NMR analysis.

### 2.5. Sample Preparation

Urine sample (440 *μ*L) was mixed with 220 *μ*L of PBS (0.2 M K_2_HPO_4_/NaH_2_PO_4_, pH 7.4) to minimize chemical shift variation because of the differences in urine pH. The samples were centrifuged at 4000 g for 5 min to remove any precipitates. The supernatant (500 *μ*L) was transferred into 5 mm NMR tubes, to which 50 *μ*L of TSP (1 mg/mL) acting as the chemical shift reference (*δ* 0.0 ppm) and 20 *μ*L of D_2_O providing a lock signal were added.

Intracellular metabolites were extracted by the modified extraction protocol as previously described [18]. The dried cell pellet was briefly homogenized and extracted in 0.80 mL of methanol for 20 min, 0.8 mL of chloroform for 15 min, and 0.72 mL of water for 20 min. The mixture was then centrifuged at 10000 g for 15 min to produce a biphasic mixture from which the aqueous and organic phases were separately removed with a pipette. The extraction solvents were completely removed by vacuum. Each polar sample was reconstituted in 40 *μ*L of phosphate buffer (100 mM Na_2_HPO_4_/NaH_2_PO_4_, including 0.5 mM TSP, pH 7.0) in D_2_O, and each lipophilic sample was reconstituted in 60 *μ*L of CDCl_3_ containing 1.0 mM tetramethylsilane (TMS) for NMR analysis.

### 2.6. NMR Spectroscopy

The ^1^H-NMR spectra of rat urine and cell samples were acquired on a Bruker 400 MHz spectrometer at 300 K by using a 5 mm conventional probe or 1.7 mm TXI microprobe. A total of 5 min were allowed for the thermal equilibration before NMR acquisition. 1D spectra were acquired for the urine samples and polar metabolites of HepG2 cells by using a standard NOESY pulse sequence with 128 transients, 100 ms mixing time, and 3.0 s relaxation delay. A single pulse experiment was used to the apolar metabolites of HepG2 cells [19]. All ^1^H NMR spectra were corrected for phase and baseline distortions and referenced to the internal reference standard TSP or TMS (*δ*_1H_ = 0.0 ppm).

### 2.7. NMR Data Analysis

All 1D NMR spectra were processed with 0.3 Hz apodization followed by zero filling to 128 k points. Each ^1^H NMR spectrum was segmented into 0.005 ppm bins, corresponding to the regions *δ* 0.2–10.0 ppm and *δ* 0.2–6.0 ppm in polar and apolar samples, respectively, by using MestReNova 6.0 (Mestrelab Research S.L.). The intensity data of water (*δ* 4.6–5.0 ppm) were excluded prior to analysis. The total spectral area of the remaining bins was normalized to unit total intensity.

The data of binned spectra were imported into SIMCA-P 13.0 demo (Umetrics Inc., Umea, Sweden) for pattern recognition analysis. Principal components analysis (PCA), an unsupervised pattern recognition method, was applied to identify general metabolic trends and possible outliers. The orthogonal partial least squared discriminant analysis (OPLS-DA) algorithm at Pareto scaling approach was used to find the main changing metabolites related to matrine exposures [20]. Subsequently, the distinguishable peak area of the selected metabolites from the NMR was extracted (normalized to the total spectra area) to further evaluate the time course and dose dependence of matrine-related metabolite variations.

### 2.8. Statistical Methods

Statistical analyses were performed using SPSS software version 11.5. The potential biomarkers were analyzed using single factor ANOVA, followed by the* post hoc* Dunnett's T3 test for nonhomogeneous variance or the Dunnett test for heterogeneity variance. *p* < 0.05 was considered statistically significant.

## 3. Results

### 3.1. Metabolite Identification in Rat Urine and HepG2 Cells by ^1^H NMR

**Figure 1:**
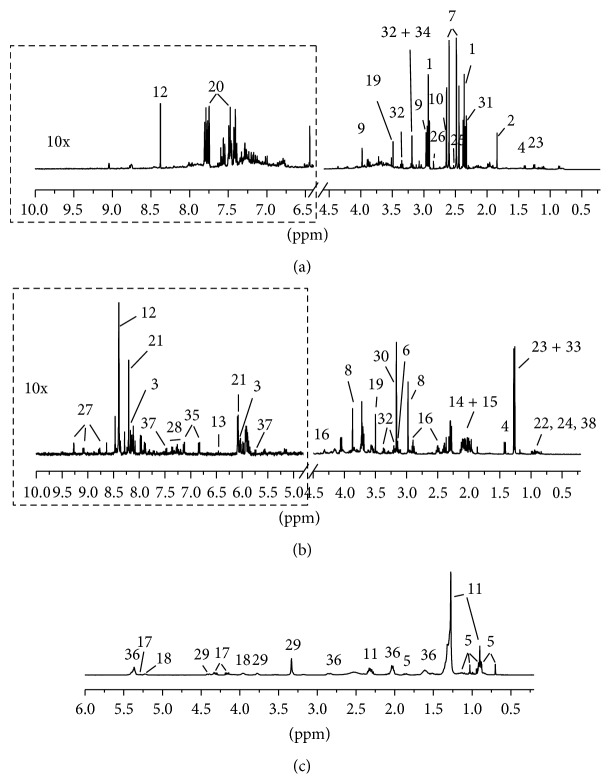
Representative ^1^H NMR spectra of control groups for (a) urine of rat and (b) aqueous and (c) lipophilic extracts of HepG2 cells. The aromatic regions (in the dashed boxes) were magnified 10 times compared with corresponding aliphatic regions for the purpose of clarity. The metabolites numbered were assigned in Table 1.

Figure 1 shows the representative ^1^H NMR spectra acquired using the rat urine (Figure 1(a)) and HepG2 cells (aqueous extracts, Figure 1(b), and lipophilic extracts, Figure 1(c)) of a control experimental subject from this study with some resonance assignments indicated. The ^1^H chemical shift assignments for metabolites were created using a combination of previously reported values [21, 22], standard compounds [23, 24], and Chenomx database entries (Edmonton, Alberta, Canada). The ^1^H-NMR spectra of rat urine and HepG2 cells contained numerous resonance metabolites, including amines (dimethylamine and TMAO), several amino acids (alanine, glutamate, glutamine, glycine, tyrosine, etc.) and amino acid derivatives (creatine, taurine, and glutathione (GSH)), choline and choline-containing compounds (glycerophosphocholine, phosphocholine), lipids (fatty acid and unsaturated fatty acid), and energy storage compounds (ATP), among others. A total of about 40 metabolites in urine and HepG2 cells were identified (Table 1).

### 3.2. Metabolomic Characterization of Urine in Rats

**Figure 2:**
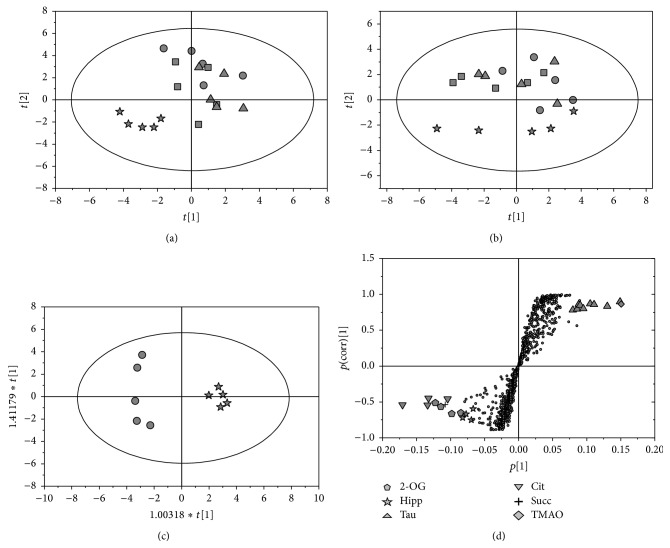
Multivariate statistical analysis based on ^1^H NMR spectra of rat urine. PCA scores plots obtained from rat urine in posttreatment day 1 (a) and day 3 (b). Representative OPLS-DA scores plot (c) and corresponding S-plot (d) derived from control group versus high dose group in posttreatment day 1. (●) control group, (▲) low dose group, (■) middle dose group, and (★) high dose group. 2-OG: 2-oxoglutarate, Cit: citrate, Hipp; hippurate, Succ: succinate, Tau: taurine, and TMAO: trimethylamine N-oxide.

**Figure 3:**
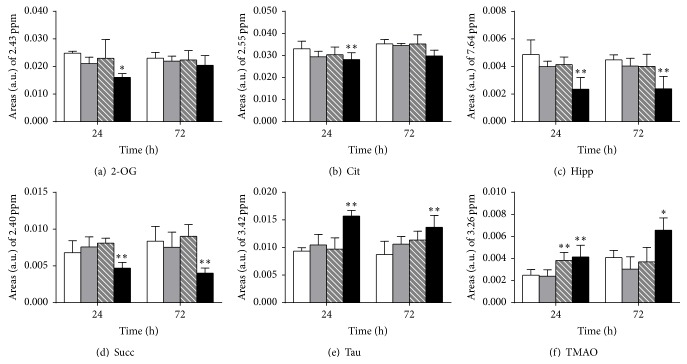
Abundance of selected metabolites was obtained by measuring the integral of a single, resolved resonance from each metabolite in ^1^H-NMR of urine from rats after treatment with matrine. (a) 2-oxoglutarate, (b) citrate, (c) hippurate, (d) succinate, (e) taurine, (f) trimethylamine N-oxide. White bar: control group, gray bar: low dose group, striped bar: middle dose group, and black bar: high dose group. Data presented in this figure were mean ± SD (*n* = 5). Asterisks indicate statistically significant differences between control and treated cells (^*∗*^ *p* < 0.05, ^*∗∗*^ *p* < 0.01).

PCA was applied to the corresponding spectra to determine the extent of differences between the treated and control groups. As shown in Figures 2(a) and 2(b), the PCA score plots of urine samples demonstrate a clear discrimination of high dose groups of control rats, at both 24 and 72 h after dose, whereas the low dose classes overlapped with the controls at the two time points. Subsequently, the OPLS-DA models (Figure 2(c)) were conducted on the corresponding NMR data from the pairwise rat urine (control versus each matrine treatment). The prominent changes in the endogenous urinary metabolites comprised a decrease in the levels of succinate, 2-oxoglutarate, citrate, and hippurate. By contrast, an elevation in taurine and TMAO was achieved by the S-plot of the OPLS-DA analysis (Figure 2(d)). Finally, these endogenous metabolites were selected as putative biomarkers, and the relative levels were calculated by measuring the peak area of a resolved resonance for each component (Figure 3). The decrease in 2-oxoglutarate, citrate, succinate, and hippurate and the increase in taurine and TMAO show pronounced dose-response trends, particularly noted for high dose treatment.

### 3.3. Metabolomic Characterization of HepG2 Cells

**Figure 4:**
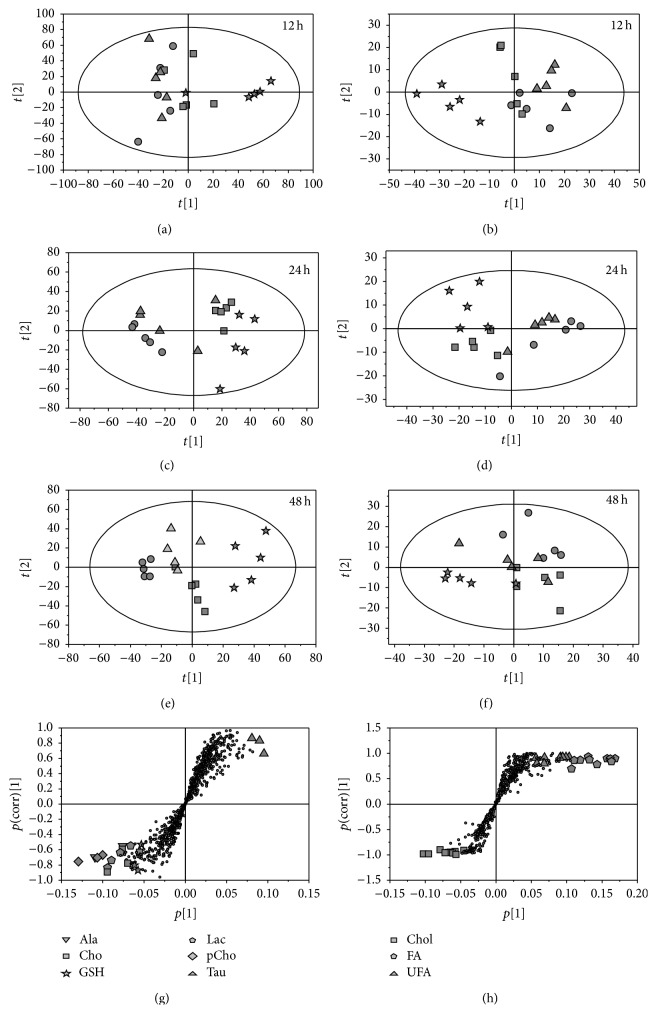
Multivariate statistical analysis based on ^1^H NMR spectra of HepG2 cells. PCA scores plots obtained from aqueous (a, c, and e) and lipophilic extracts (b, d, and f) of HepG2 cells following exposure to matrine for 12, 24, and 48 h, respectively. Representative S-plots of the OPLS-DA model derived from ^1^H-NMR data for aqueous (g) and lipophilic (h) extracts of HepG2 cells after the exposure to high dose matrine for 48 h. (●) control group, (▲) low dose group, (■) middle dose group, and (★) high dose group. Ala: alanine, Cho: choline, GSH: glutathione, Lac: lactate, pCho: phosphocholine, Tau: taurine, Chol: cholesterol, FA: fatty acid, and UFA: unsaturated fatty acid.

**Figure 5:**
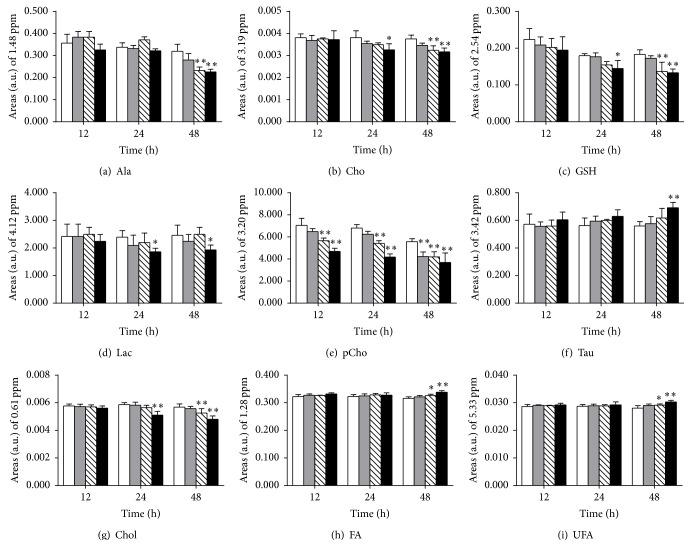
Abundance of selected metabolites was obtained by measuring the integral of a single, resolved resonance from each metabolite in ^1^H-NMR of HepG2 cells upon exposure time. (a) Alanine, (b) choline, (c) glutathione, (d) lactate, (e) phosphocholine, (f) taurine, (g) cholesterol, (h) fatty acid, and (i) unsaturated fatty acid. White bar: control group, gray bar: low dose group, striped bar: middle dose group, and black bar: high dose group. Data presented in this figure were mean ± SD (*n* = 5). Asterisks indicate statistically significant differences between control and treated cells (^*∗*^ *p* < 0.05, ^*∗∗*^ *p* < 0.01).

The PCA score plots revealed that low dose groups overlapped with their controls at 12 and 24 h and removed from the control group prolonging the cultured time to 48 h, in both aqueous extracts (Figures 4(a), 4(c), and 4(e)) and lipophilic extracts (Figures 4(b), 4(d), and 4(f)) of HepG2 cells. By contrast, the high dose groups were far from their controls at all-time points, and the middle groups were between the two clusters. OPLS-DA analysis was conducted on the corresponding NMR data from the pairwise cell groups (control versus each matrine administration group) to investigate the metabolic markers of HepG2 cell response to matrine. Compared with their control groups, the significant metabolic responses (Figures 4(g) and 4(h)) were selected as potential biomarkers, and the relative concentration was calculated using the peak area as the methods of urine samples. From the plot of relative levels of selected metabolites in HepG2 cells (Figure 5), taurine, fatty acid, and unsaturated fatty acid clearly increased, whereas alanine, choline, GSH, lactate, phosphocholine, and cholesterol decreased in 48 h high dose group. In addition, these metabolites also showed a significant change in 24 h high dose and/or 48 h middle dose group but did not significantly change in the low dose groups at different time periods, except the evident decrease of phosphocholine prolonging the cultured time to 48 h.

## 4. Discussion

The relative contents of 2-oxoglutarate, citrate, and succinate in the urine of SD rats significantly decreased in response to matrine treatment. These metabolites are tricarboxylic acid cycle intermediates known to be involved in energy metabolism. The decreases in the relative contents of these metabolites in the urine of high dose matrine-treated rats reveal the inhibition of energy metabolism by this drug.

Taurine, an essential amino acid present in various mammalian tissues [25], possesses various diverse biological functions, such as transmission of nerve impulses, modulation of calcium signaling, and membrane stabilization. Increased taurine in urine is a biomarker of liver damage induced by several drugs [21, 26, 27]. Therefore, the rise in taurine level in the rat urine reveals liver dysfunction and injury after treatment with high dose matrine.

TMAO, an oxidation product of trimethylamine, is converted from choline. TMAO is a major osmolyte that the body uses to counteract the effects of increased urea concentrations, which is due to kidney failure. High levels of TMAO can be used as a biomarker for kidney problems [25, 28, 29]. In this study, increased TMAO levels were noted in the urine of treated rats as dose dependent, suggesting that the osmotic dysregulation in renal tissue is due to matrine-induced toxicity.

Hippurate, also known as acyl glycine, is formed by the conjugation of benzoic acid with glycine, which is catalyzed by glycine N-acyltransferase [30]. Hippurate is a normal component of urine and typically increases with increased consumption of phenolic compounds; these compounds are converted to benzoic acid and then to hippurate and subsequently excreted in the urine. Thus, Um et al. [31] reported that low hippurate levels are found in indomethacin-induced gastrointestinal damage, which may break normal microorganism balance in the GI tract. Hippurate concentration also decreases in the rat urine after using antibiotics to sterilize the intestine [32]. Therefore, the decrease in hippurate levels after treatment with matrine may correlate with disturbance in the gut microbiota.

Lactate, the end product of glycolysis, has been reported to increase in several cancers [33–36], in consequence, of the Warburg effect. Alanine is released from the alanine-glucose cycle after pyruvate formation from glycolysis [37]. Several studies have reported decreased lactate and alanine levels in tumor cells in response to chemotherapy drugs [38–41], thereby reducing the glycolysis rate. In this study, lactate and alanine concentrations were decreased in aqueous extraction of HepG2 cells in a time- and dose-dependent manner, indicating that matrine is less anaerobic in HepG2 cells

The lipid signals of HepG2 cells significantly changed in response to matrine treatment, which is similar to the findings that lipids increase in tumor cells in response to chemotherapy drugs [22, 42]. The lipid signals in the NMR spectrum have been suggested to originate from the fatty acyl chain of lipid droplets in the cytosol but not from the membrane lipids [42]. Lipid droplets perform various biological functions in cells, such as energy storage, providing phospholipids and sterols for the synthesis of biological membranes, and buffer cells from the toxic effects of excessive amounts of lipid [43]. Lipid droplets have been recently identified as a substrate for macroautophagy, which regulates a number of essential cellular processes, including development and differentiation, immunity, apoptosis, and aging; these droplets also represent a new cellular target for abnormalities in lipid metabolism and accumulation [44]. Although the autophagy characteristics of HepG2 cells in this study were not monitored, the accumulation of autophagic vacuoles in matrine-treated cells has been verified in human hepatoma G2 cells [45], gastric cancer SGC-7901 cells (10), and rat C6 glioma cells [46]. These findings suggested a possible link between the increase of lipids and matrine-affected autophagy and may be related to the apoptosis regulation of HepG2 cells (IC_50_ was 0.71 mg/mL, data not shown).

GSH, also known as g-L-glutamyl-L-cysteine-glycine, is a ubiquitous tripeptide that functions as a cellular thiol “redox buffer” to maintain a given thiol/disulfide redox potential. A possible link among thiol redox state, glutathione-protein interactions, and cell proliferation has been reported [47]. In this study, an evident decrease in intracellular GSH levels in HepG2 cells was detected after exposure to matrine, which agrees with the result of glutathione reductase measurement [48], and it reflected the change of the redox state in cells.

Phosphocholine and choline decrease in response to matrine exposure in a dose- and time-dependent manner, suggesting that matrine induced the changes of phospholipid biosynthesis or PTC cycle regulation in HepG2 cells. The induction of this effect has been demonstrated by other chemotherapeutic drugs [49]. The decrease in cholesterol levels in the high dose matrine-treated group indicates decreased cholesterogenesis [41]. In addition, taurine concentration is increased after cell exposure to matrine. Similar results have also been observed in HepG2 cells treated with TCDD [31] and as an* in vivo* biomarker of apoptosis in glial tumors [50]. Therefore, the increased taurine in HepG2 cells may have been related to apoptosis induced by matrine.

## 5. Conclusions

Higher dose of matrine has an evident toxic potential both* in vivo* and* in vitro*. Treatment with matrine caused dysfunction of energy metabolism in SD rats and HepG2 cells. In addition, the high dose of matrine also affected the liver, renal function, and gut microbiota in rats and lipid metabolism, redox state, phospholipid biosynthesis or PTC cycle, and cholesterogenesis in HepG2 cells. Few metabolites including 2-oxoglutarate, citrate, succinate, hippurate, taurine, and TMAO in rat urine, as well as alanine, choline, glutathione, lactate, phosphocholine, taurine, cholesterol, fatty acid, and unsaturated fatty acid in HepG2 cells, are potential markers of matrine use. These metabolites may be used to facilitate the diagnosis of matrine-induced toxicity.

## Figures and Tables

**Table 1:** The ^1^H NMR data of the metabolites in the urine of rats and HepG2 cells.

No.	Metabolites	^1^H Shift (multiplicity)	Sample
1^A^	2-oxoglutarate	2.43 (t^B^), 3.00 (t)	U^C^
2	Acetate	1.92 (s)	U, C_a_
3	ADP/ATP	5.99 (s), 7.88 (d), 8.19 (s)	C_a_
4	Alanine	1.48 (d), 3.78 (q)	U, C_a_
5	Cholesterol (total)	0.61 (s), 0.79 (dd), 0.84 (d), 0.94 (s), 1.76 (m)	C_l_
6	Choline	3.19 (s)	C_a_
7	Citrate	2.55 (d), 2.70 (d)	U
8	Creatine	3.04 (s), 3.93 (s)	C_a_
9	Creatinine	3.04 (s), 4.06 (s)	U
10	Dimethylamine	2.72 (s)	U
11	Fatty Acid	0.81 (t), 1.20 (m), 2.24 (m)	C_l_
12	Formate	8.46 (s)	U, C_a_
13	Fumarate	6.52 (s)	C_a_
14	Glutamate	2.10 (m), 2.35 (m), 3.78 (t)	C_a_
15	Glutamine	2.11 (m), 2.44 (m), 3.77 (t)	C_a_
16	Glutathione	2.54 (m), 2.95 (m), 4.56 (m)	C_a_
17	Glycerol	4.07 (dd), 4.22 (dd), 5.19 (br)	C_l_
18	Glycerophospholipid	3.86 (s), 5.13 (m)	C_l_
19	Glycine	3.56 (s)	U, C_a_
20	Hippurate	7.83 (d), 7.64 (t), 7.55 (t), 3.97 (d)	U
21	Inosine	8.22 (s), 8.33 (s)	C_a_
22	Isoleucine	0.94 (t), 1.01 (d)	C_a_
23	Lactate	1.33 (d), 4.12 (q)	U, C_a_
24	Leucine	0.96 (d), 0.97 (d)	C_a_
25	Methylamine	2.61 (s)	U
26	N,N-Dimethylglycine	2.92 (s)	U
27	NADP+	8.84 (d), 9.14 (d), 9.33 (s)	C_a_
28	Phenylalanine	7.33 (d), 7.38 (t), 7.43 (m)	C_a_
29	Phosphatidylcholine	3.23 (s), 3.67 (s), 4.32 (m)	C_l_
30	Phosphocholine	3.20 (s)	C_a_
31	Succinate	2.40 (s)	U
32	Taurine	3.25 (t), 3.42 (t)	U, C_a_
33	Threonine	1.33 (d)	C_a_
34	Trimethyamine N-oxide	3.26 (s)	U
35	Tyrosine	6.92 (d), 7.20 (d)	C_a_
36	Unsaturated Fatty Acid	2.75 (m), 5.27 (br)	C_l_
37	Uracil	5.79 (d), 7.52 (d)	C_a_
38	Valine	0.99 (d), 1.04 (d)	C_a_

^A^The numbering system is the same as used in Figure 1.

^B^Multiplicity: s, singlet; d, doublet; t, triplet; q, quartet; dd, doublet of doublets; m, multiplet; br, broad resonance.

^C^U, urine of rats; C_a_, aqueous extracts of HepG2 cells; C_l_, lipophilic extracts of HepG2 cells.

## References

[1] Li X.-M., Brown L. (2009). Efficacy and mechanisms of action of traditional Chinese medicines for treating asthma and allergy. *Journal of Allergy and Clinical Immunology*.

[2] Guzman J. R., Koo J. S., Goldsmith J. R., Mühlbauer M., Narula A., Jobin C. (2013). Oxymatrine prevents NF-*κ*B nuclear translocation and ameliorates acute intestinal inflammation. *Scientific Reports*.

[3] Yang Y., Xiu J., Zhang X. (2012). Antiviral effect of matrine against human enterovirus 71. *Molecules*.

[4] Liu Y., Xu Y., Ji W. (2014). Anti-tumor activities of matrine and oxymatrine: literature review. *Tumor Biology*.

[5] Sun M., Cao H., Sun L. (2012). Antitumor activities of kushen: literature review. *Evidence-Based Complementary and Alternative Medicine*.

[6] Zhou H., Xu M., Gao Y. (2014). Matrine induces caspase-independent program cell death in hepatocellular carcinoma through bid-mediated nuclear translocation of apoptosis inducing factor. *Molecular Cancer*.

[7] Liu G. Q., Yuan H. N., Xie L., Jin X. N., Lid X. Q. (1987). Effect of sophocarpine and other alkaloids from *Sophora alopeculoides* L. on monoamine metabolism, dopamine and 5-HT receptors. *Yao Xue Xue Bao*.

[8] Lu Z.-G., Li M.-H., Wang J.-S., Wei D.-D., Liu Q.-W., Kong L.-Y. (2014). Developmental toxicity and neurotoxicity of two matrine-type alkaloids, matrine and sophocarpine, in zebrafish (Danio rerio) embryos/larvae. *Reproductive Toxicology*.

[9] Wang X.-Y., Liang L., Chang J.-L., Yang M.-H., Li Z.-G. (2010). Toxicity of matrine in Kunming mice. *Nan Fang Yi Ke Da Xue Xue Bao*.

[10] Drew A. K., Bensoussan A., Whyte I. M., Dawson A. H., Zhu X., Myers S. P. (2002). Chinese herbal medicine toxicology database: monograph on radix sophorae flavescentis, ‘Ku Shen’. *Journal of Toxicology—Clinical Toxicology*.

[11] Wang X. P., Yang R. M. (2003). Movement disorders possibly induced by traditional Chinese herbs. *European Neurology*.

[12] Čuperlović-Culf M., Barnett D. A., Culf A. S., Chute I. (2010). Cell culture metabolomics: applications and future directions. *Drug Discovery Today*.

[13] Boudonck K. J., Mitchell M. W., Német L. (2009). Discovery of metabolomics biomarkers for early detection of nephrotoxicity. *Toxicologic Pathology*.

[14] Boudonck K. J., Rose D. J., Karoly E. D., Lee D. P., Lawton K. A., Lapinskas P. J. (2009). Metabolomics for early detection of drug-induced kidney injury: review of the current status. *Bioanalysis*.

[15] Ni Y., Xie G., Jia W. (2014). Metabonomics of human colorectal cancer: new approaches for early diagnosis and biomarker discovery. *Journal of Proteome Research*.

[16] Holmes E., Bonner F. W., Nicholson J. K. (1995). Comparative studies on the nephrotoxicity of 2-bromoethanamine hydrobromide in the Fischer 344 rat and the multimammate desert mouse (*Mastomys natalensis*). *Archives of Toxicology*.

[17] Kim J. W., Ryu S. H., Kim S. (2013). Pattern recognition analysis for hepatotoxicity induced by acetaminophen using plasma and urinary 1H NMR-based metabolomics in humans. *Analytical Chemistry*.

[18] Teng Q., Huang W., Collette T. W., Ekman D. R., Tan C. (2009). A direct cell quenching method for cell-culture based metabolomics. *Metabolomics*.

[19] Bringaud F., Biran M., Millerioux Y., Wargnies M., Allmann S., Mazet M. (2015). Combining reverse genetics and nuclear magnetic resonance-based metabolomics unravels trypanosome-specific metabolic pathways. *Molecular Microbiology*.

[20] Wiklund S., Johansson E., Sjöström L. (2008). Visualization of GC/TOF-MS-based metabolomics data for identification of biochemically interesting compounds using OPLS class models. *Analytical Chemistry*.

[21] Wang H., Bai J., Chen G. (2013). A metabolic profiling analysis of the acute hepatotoxicity and nephrotoxicity of Zhusha Anshen Wan compared with cinnabar in rats using ^1^H NMR spectroscopy. *Journal of Ethnopharmacology*.

[22] Ruiz-Aracama A., Peijnenburg A., Kleinjans J. (2011). An untargeted multi-technique metabolomics approach to studying intracellular metabolites of HepG2 cells exposed to 2,3,7,8-tetrachlorodibenzo-p-dioxin. *BMC Genomics*.

[23] Ulrich E. L., Akutsu H., Doreleijers J. F. (2008). BioMagResBank. *Nucleic Acids Research*.

[24] Wishart D. S., Jewison T., Guo A. C. (2013). HMDB 3.0—the human metabolome database in 2013. *Nucleic Acids Research*.

[25] Fukuda K., Hirai Y., Yoshida H., Nakajima T., Usui T. (1982). Free amino acid content of lymphocytes and granulocytes compared. *Clinical Chemistry*.

[26] Li X., Zhang F., Wang D., Li Z., Qin X., Du G. (2014). NMR-based metabonomic and quantitative real-time PCR in the profiling of metabolic changes in carbon tetrachloride-induced rat liver injury. *Journal of Pharmaceutical and Biomedical Analysis*.

[27] Waterfield C. J., Turton J. A., Scales M. D. C., Timbrell J. A. (1993). Investigations into the effects of various hepatotoxic compounds on urinary and liver taurine levels in rats. *Archives of Toxicology*.

[28] Gartland K. P. R., Bonner F. W., Nicholson J. K. (1989). Investigations into the biochemical effects of region-specific nephrotoxins. *Molecular Pharmacology*.

[29] Foxall P. J. D., Mellotte G. J., Bending M. R., Lindon J. C., Nicholson J. K. (1993). NMR spectroscopy as a novel approach to the monitoring of renal transplant function. *Kidney International*.

[30] Phipps A. N., Stewart J., Wright B., Wilson I. D. (1998). Effect of diet on the urinary excretion of hippuric acid and other dietary-derived aromatics in rat. A complex interaction between diet, gut microflora and substrate specificity. *Xenobiotica*.

[31] Um S. Y., Park J. H., Chung M. W. (2012). Nuclear magnetic resonance-based metabolomics for prediction of gastric damage induced by indomethacin in rats. *Analytica Chimica Acta*.

[32] Williams R. E., Eyton-Jones H. W., Farnworth M. J., Gallagher R., Provan W. M. (2002). Effect of intestinal microflora on the urinary metabolic profile of rats: a 1H-nuclear magnetic resonance spectroscopy study. *Xenobiotica*.

[33] Draoui N., Feron O. (2011). Lactate shuttles at a glance: from physiological paradigms to anti-cancer treatments. *Disease Models and Mechanisms*.

[34] Gatenby R. A., Gillies R. J. (2004). Why do cancers have high aerobic glycolysis?. *Nature Reviews Cancer*.

[35] Engskog M. K. R., Björklund M., Haglöf J., Arvidsson T., Shoshan M., Pettersson C. (2015). Metabolic profiling of epithelial ovarian cancer cell lines: evaluation of harvesting protocols for profiling using NMR spectroscopy. *Bioanalysis*.

[36] Whitaker-Menezes D., Martinez-Outschoorn U. E., Lin Z. (2011). Evidence for a stromal-epithelial ‘lactate shuttle’ in human tumors: MCT4 is a marker of oxidative stress in cancer-associated fibroblasts. *Cell Cycle*.

[37] Louis P., Hold G. L., Flint H. J. (2014). The gut microbiota, bacterial metabolites and colorectal cancer. *Nature reviews. Microbiology*.

[38] Belt J. A., Thomas J. A., Buchsbaum R. N., Racker E. (1979). Inhibition of lactate transport and glycolysis in Ehrlich ascites tumor cells by bioflavonoids. *Biochemistry*.

[39] Klawitter J., Anderson N., Klawitter J. (2009). Time-dependent effects of imatinib in human leukaemia cells: a kinetic NMR-profiling study. *British Journal of Cancer*.

[40] Le A., Cooper C. R., Gouw A. M. (2010). Inhibition of lactate dehydrogenase A induces oxidative stress and inhibits tumor progression. *Proceedings of the National Academy of Sciences of the United States of America*.

[41] Raina K., Serkova N. J., Agarwal R. (2009). Silibinin feeding alters the metabolic profile in TRAMP prostatic tumors: 1H-NMRS-based metabolomics study. *Cancer Research*.

[42] Duarte I. F., Ladeirinha A. F., Lamego I. (2013). Potential markers of cisplatin treatment response unveiled by NMR metabolomics of human lung cells. *Molecular Pharmaceutics*.

[43] Farese R. V., Walther T. C. (2009). Lipid droplets finally get a little R-E-S-P-E-C-T. *Cell*.

[44] Rambold A., Cohen S., Lippincott-Schwartz J. (2015). Fatty acid trafficking in starved cells: regulation by lipid droplet lipolysis, autophagy, and mitochondrial fusion dynamics. *Developmental Cell*.

[45] Zhang J.-Q., Li Y.-M., Liu T. (2010). Antitumor effect of matrine in human hepatoma G2 cells by inducing apoptosis and autophagy. *World Journal of Gastroenterology*.

[46] Zhang S., Qi J., Sun L. (2009). Matrine induces programmed cell death and regulates expression of relevant genes based on PCR array analysis in C6 glioma cells. *Molecular Biology Reports*.

[47] Dröge W., Hack V., Breitkreutz R. (1998). Role of cysteine and glutathione in signal transduction, immunopathology and cachexia. *BioFactors*.

[48] Cheng X., Du Y., Huang L., Jing Z., Zheng Z. (2008). Effect of matrine on HepG2 cells: role of glutathione and cytochrome c. *The Chinese-German Journal of Clinical Oncology*.

[49] Duarte I. F., Lamego I., Marques J., Marques M. P. M., Blaise B. J., Gil A. M. (2010). Nuclear magnetic resonance (NMR) study of the effect of cisplatin on the metabolic profile of MG-63 osteosarcoma cells. *Journal of Proteome Research*.

[50] Opstad K. S., Bell B. A., Griffiths J. R., Howe F. A. (2009). Taurine: a potential marker of apoptosis in gliomas. *British Journal of Cancer*.

